# Atypical identification of Lynch syndrome by immunohistochemistry and microsatellite instability analysis on jejunal adenocarcinoma

**DOI:** 10.1186/1897-4287-9-S1-P27

**Published:** 2011-03-10

**Authors:** Dawn E McIlvried, Ruemu E Birhiray, Jim Z Lu

**Affiliations:** 1 Department of Cancer Genetics, St. Vincent Hospital, Indianapolis, IN 46260, USA; 2 Hematology Oncology of Indiana, PC, Indianapolis, IN 46260, USA; 3 AmeriPath® Indiana, Indianapolis, IN 46219, USA

## Background

Lynch syndrome (LS) is a hereditary cancer condition associated with germline mutations in the mismatch repair genes MLH1, MSH2, MSH6 and PMS2. This condition has been traditionally described in terms of an increased risk for early-onset colorectal and endometrial cancers. Individuals with LS additionally have an increased risk to develop other cancers including ovarian, gastric, urinary tract, hepatobiliary tract, small bowel, brain, and skin cancers. The preferred method of genetic testing for LS is to initially perform microsatellite instability (MSI) analysis and/or immunohistochemistry (IHC) staining as a test on the tumor tissue of an affected individual. If abnormal, this testing would indicate that a germline mismatch repair gene mutation is more likely to explain the cancer and, if IHC is performed, may also indicate which gene to test first via molecular analysis. MSI/IHC has been more widely validated for colon and endometrial cancers as opposed to other cancers associated with this condition.

## Case report

**Figure 1:**
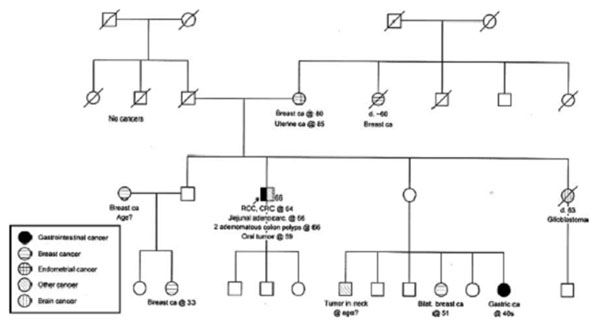
Pedigree of Lynch syndrome family with MSH6 mutation

**Figure 2:**
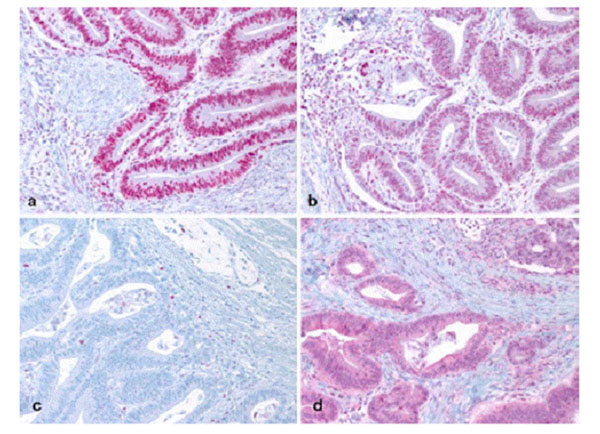
Immunihistochemistry on jejunal adenocarcinoma demonstrating loss of MSH6 protein expression. Immunohistochemical staining of patient small bowel adenocarcinoma tissue demonstrating normal protein expression for MLH1 (a), MSH2 (b), and PMS2 (d), and abnormal lack of expression for MSH6 (c). Note: Internal control tissue was evaluated and revealed positive staining while tumor cells were negative.

We report a 66-year-old male patient with past history of multiple cancers including colon and kidney cancer, and recently diagnosed with jejunal adenocarcinoma. Family history of Lynch-associated cancers included brain, gastric and endometrial cancer, noted to have later ages of onset (Figure 1). The patient had MSI/IHC testing performed on his small bowel cancer and this was reported as abnormal with MSI-high in 5/5 markers and absent MSH6 staining (Figure 2). Subsequent blood draw revealed the presence of a pathogenic MSH6 gene mutation, 3202C>T (R1068X).

## Conclusion

As we learn more about the etiology and cancer risks associated with LS, the expanding phenotypic spectrum of this condition and its genotype-phenotype correlations are being elucidated. Specifically, MSH6 mutations have been associated with a higher cumulative risk for endometrial cancer with a later age of onset as compared to other mismatch repair gene mutations [1-5], and cancers other than colon and endometrial may account for as much as 50% of cancer reported with MSH6 mutations [6]. Clarifying the efficacy of MSI/IHC in other extra-colonic cancers associated with LS is important, since certain cases of LS are likely to present in a clinically atypical manner.

This case illustrates the phenotypic variability of this condition, and complications it may present in evaluation for diagnosis and appropriate surveillance and management recommendations. It also suggests that testing by MSI/IHC may be very feasible as a first step in LS identification using tumor tissue beyond what has been traditionally reported in the literature.
